# Kirners deformity – a systematic review and surgery recommendations

**DOI:** 10.1007/s00402-024-05724-5

**Published:** 2025-01-03

**Authors:** Tim Fülling, Carsten Baade, Adrian Dragu, Antek Nicklas

**Affiliations:** 1 Abteilung für Plastische und Handchirurgie UniversitätsCentrum für Orthopädie, Unfall- & Plastische Chirurgie, Universitätsklinikum Carl Gustav Carus an der Technischen Universität Dresden, Dresden, Germany; 2 Helios Weißeritztal-Kliniken GmbH, Freital, Germany

**Keywords:** Kirners deformity, Hand surgery, Surgery recommendations, Dystelephalangy, Finger deformity

## Abstract

**Background:**

Kirner deformity is a rare anomaly of the little finger in adolescents, characterized by a deformity of the distal phalanx and a radiologically L-shaped epiphysis, along with palmar and radial angulation of the distal phalanx. Due to the rarity of these pathological findings, there are no systematic literature reviews available. This work serves as an overview of the clinical presentation, frequency and age distributions, as well as possible conservative and surgical treatment options.

**Methods:**

We present five cases of patients with Kirner’s deformity of the little finger who underwent surgical treatment. A partial tenotomy of the flexor digitorum profundus tendon from the metaphyseal/diaphyseal distal phalanx was performed. In one case, a dorsal epiphysiodesis was also carried out. Additionally, a systematic review of the literature on Kirner’s deformity was conducted, summarizing the prevalence, previously used surgical treatment options, and epidemiological data.

**Results:**

In the presented cases, the detachment of the FDP tendon and dorsal epiphysiodesis resulted in a good functional and aesthetic outcome. Regarding the epidemiological distribution of Kirner deformity, it is noted that significantly more females are affected than males (63% vs. 36%). The average age at presentation in the respective clinic was 9.36 years (± 2.5). In more than half of all reported cases, the deformity was bilateral. Surgical intervention was performed in only 7.4% of cases, which included FDP detachment or corrective osteotomies. More than 90% of patients were treated conservatively.

**Conclusion:**

Kirner’s deformity is a rare condition affecting adolescents. In cases where functional limitations or pain symptoms are present, we recommend surgical intervention via detachment of the FDP tendon. If the deformity is an incidental finding without functional or aesthetic limitations, conservative therapy with a corrective splint can be initiated. From our perspective, early surgical treatment before the age of 12 improves both the long-term functional and aesthetic outcomes.

## Introduction

The Kirner deformity, first described by Kirner in 1927, is a rare handsurgical anomaly that primarily affects children and adolescents [1]. It is characterized by a curvature of the distal phalanx of the fifth finger. Due to the rarity of this deformity and the limited scientific literature available, a detailed investigation is necessary to improve understanding and treatment.

### Clinical features

Kirner deformity is typically diagnosed in childhood or early adolescence and usually affects both hands. The main characteristics include a visible and palpable curvature of the distal phalanx of the little finger, which is often painless but may be cosmetically concerning. The condition is generally progressive, with the deformity worsening as the child grows.

### Radiological findings

Radiologically, the deformity presents as a palmar and radial curvature of the distal phalanx of the little finger. In advanced cases, cortical thickening and sclerotic changes may also be observed [2, 3].

### Etiology

The exact cause of Kirner deformity is unknown. However, genetic factors are suspected to play a role, as familial clustering has been reported [4, 5]. A familial inheritance (autosomal dominant) has been described^4^. Developmental disturbances of bone structure during growth may also contribute to the formation of this anomaly [6].

### Diagnostic methods

The diagnosis of Kirner deformity is primarily made through clinical examination and radiological imaging. X-rays of the affected fingers are the preferred method to visualize the characteristic curvatures and any potential structural bone changes [3].

### Differential diagnoses

It is important to differentiate Kirner deformity from other conditions that may present with similar clinical and radiological features. These include Klippel-Feil syndrome, Apert syndrome, Enchondromatosis and Clinodactyly [7].

Since injuries to the terminal phalanx are common in children, it is also important to consider fractures and pseudarthroses of the terminal phalanx as important differential diagnoses [8].

### Therapy

In most cases, no intervention is necessary, as the deformity is usually asymptomatic and does not cause functional impairments. For significant cosmetic or functional concerns, surgical correction may be considered. Surgical options include osteotomies to straighten the affected phalanx [4, 9]. Conservative approaches include physical therapy and the use of splints to slow the progression of the deformity. However, these methods have limited efficacy and are rarely employed. Surgical treatment is rarely necessary and is reserved for cases with severe cosmetic or functional impairments. Techniques such as wedge osteotomy to correct the curvature, followed by fixation with K-wires or screws, are possible procedures [10].

## Material and method

To gain a deeper understanding of this rare condition, we conducted an extensive literature review using PubMed and Google Scholar. The search terms “Kirner deformity” and “Dystelephalangy” identified 26 publications from 1985 to 2024 (Table 1). Two papers were excluded due to being overview articles without case presentations. We screened the remaining papers with a focus on conservative and surgical treatments of Kirner deformity. Additionally, epidemiological data such as age, gender, and affected finger or side were identified.

**Table 1:** Literature overview and performed operation

Author	Title	Cases	Surgery?
Adeb et al., 2019	Kirner’s deformity of the fifth finger: a case report	1	No
Benatar 2004	Kirner’s Deformity Treated by Distal Detachment of the Flexor Digitorum Profundus Tendon	4	Yes (FDP Detachement)
Brune et al., 2003	Kirner’s deformity of all fingers in a 5-year-old girl: soft-tissue enhancement with normal bones on contrast-enhanced MRI	1	No
Fairbank et al., 2015	The pathogenesis of Kirner’s deformity: A clinical, radiological and histological study	3	Yes (Osteotomy + Kirschner - Wire DIP-Transfixation)
Freiberg et al., 1985	Kirner’s deformity: A review of the literature and case presentation	63	No
Gamo et al., 2014	Percutaneous corrective osteotomy for Kirner’s deformity: a case report	1	Yes (percutaneous correction Osteotomy)
Gouda et al., 2010	Kirner’s deformity of little finger	1	No
Khalid et al., 2012	Kirner’s Deformity Misdiagnosed as Fracture: A Case Report	1	No
Lau et al., 2008	Reverse Kirner’s Deformity: Case Report	1	No
Lee et al., 2010	MRI findings in Kirner deformity: normal insertion of the flexor Digitorum profundus tendon without soft-tissue enhancement	1	No
Moser et al., 1996	A new method for treating the Kirner deformity with the SM-Fix phalangeal distractor	1	Yes (Osteotomy of distal phalanx + SM-Fix-Phalanx-Distractor)
Norat et al., 2008	Clinodactylies: Delta Phalanx and Kirner deformity	1	No
Satake et al., 2013	Radiographic features of Kirner’s deformity	36	No
Song & Koh, 2004	Kirner’s deformity: progressiveness and classification	1	No
Sugiura, 1989	Polytopic dystelephalangy of the fingers	1	No
Tianxao et al., 2020	Kirner’s deformity of the fifth finger A case report	1	No
Würfel et al., 1995	Die Kirner-Deformität	1	No

This study performed a comprehensive statistical analysis using the latest version of IBM SPSS Statistics to evaluate the data collected from all included publications. The retrospective evaluation was conducted in accordance with the Declaration of Helsinki.

## Case series

**Fig. 1:**
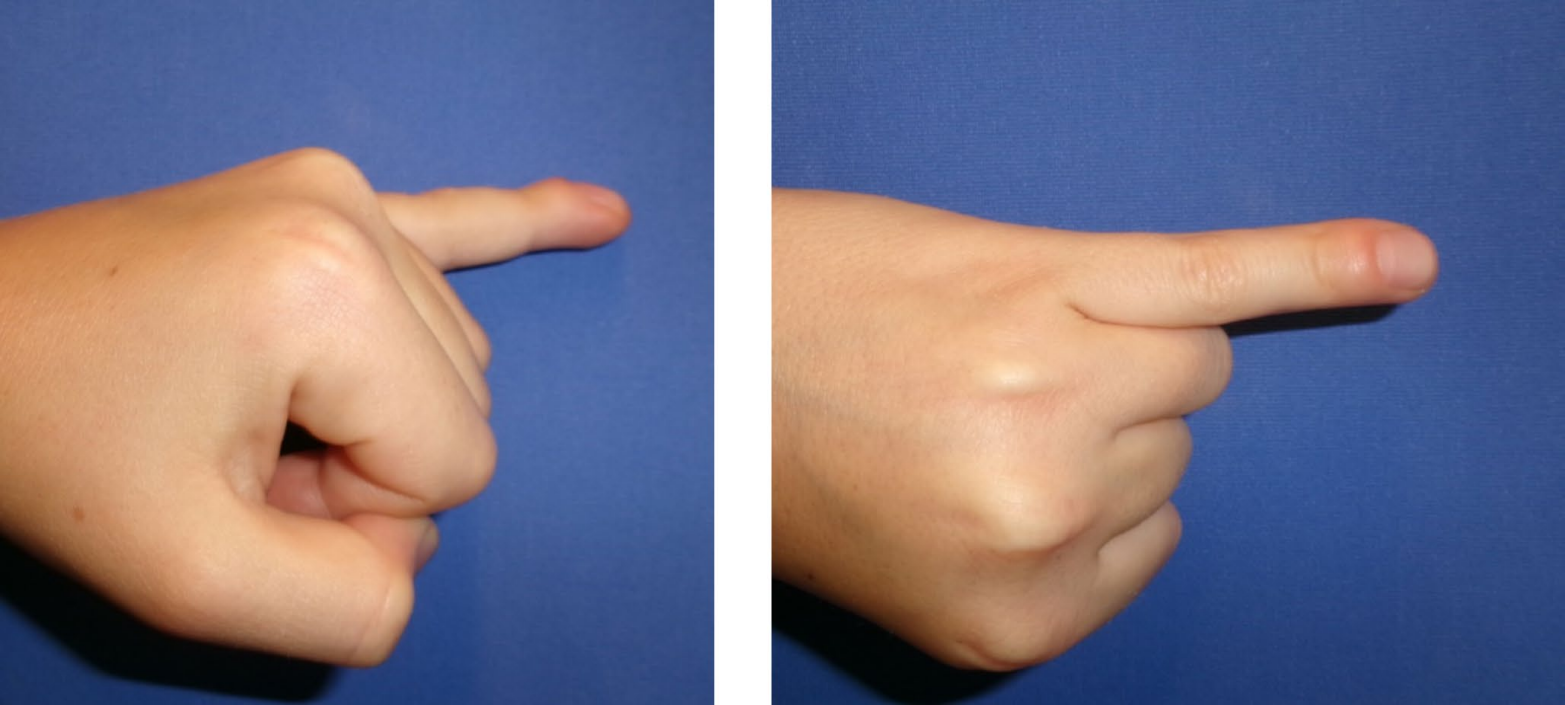
Preoperative situation; Kirners deformity of 5th finger in 12 year old patient

**Fig. 2:**
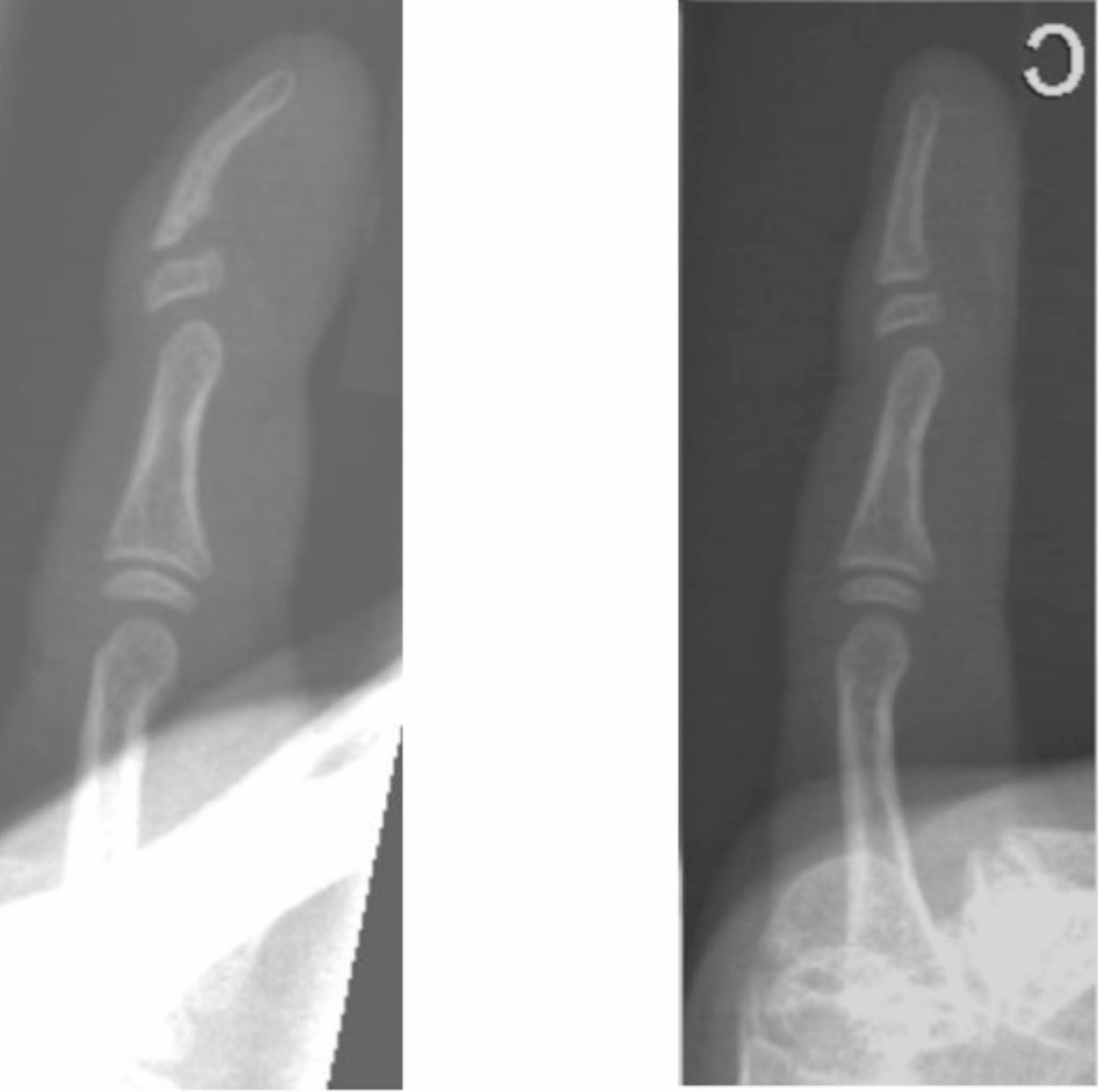
Preoperative x-ray with widened, L-shaped epiphysis with radial and palmar angulation of the distal phalanx (left); 10 months postoperative (right)

In this study, we present five cases of Kirner’s deformity of the little finger, all of which were treated surgically. Included were four female and one male patient. In three patient Kirner’s deformity occurred in the left little finger. The other patient presented with deformity in the right little finger. Clinically, all cases showed a painless deformity of the distal phalanx with radial and palmar deviation, while full mobility of the distal interphalangeal joint was preserved, with an average extension/flexion angle of 0-10-80° (Fig. 1). The joint itself was stable in each case. The X-ray of a 12-year-old patient exemplifies a widened, L-shaped epiphysis with radial and palmar angulation of the distal phalanx (Fig. 2).

**Fig. 3:**
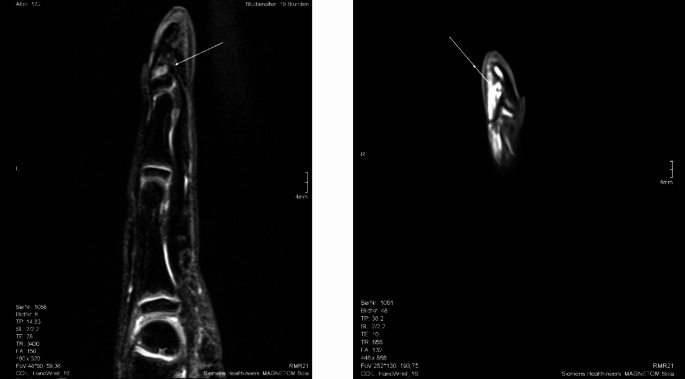
Preoperative MRI with a distally inserted flexor tendon without evidence of inflammatory activity in the epiphyseal area

**Fig. 4:**
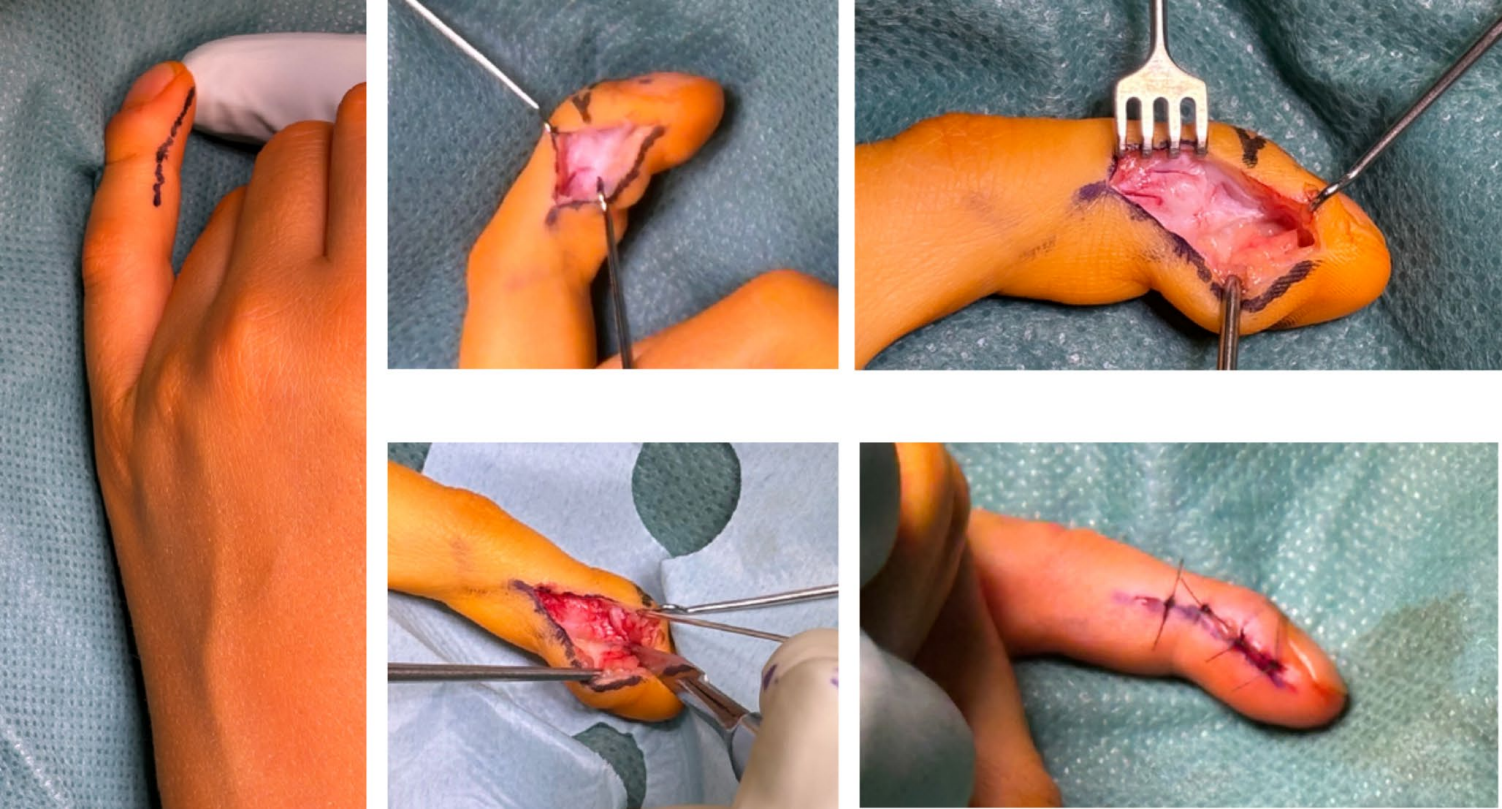
Intraoperative situs

An outpatient MRI of the same patient revealed a distally inserted flexor tendon without evidence of inflammatory activity in the epiphyseal area (Fig. 3). In all cases, we performed a partial tenotomy of the flexor digitorum profundus tendon from the metaphyseal/diaphyseal distal phalanx. In our exemplary case, a dorsal epiphysiodesis was also performed. A radial-sided incision along the neutral axis was made over the distal middle and distal phalanges. Dissection was then carried deeper to the insertion of the FDP tendon in the metaphyseal or diaphyseal region. The tendon insertion was dissected with an 11-blade (Fig. 4). Postoperatively, immobilization was achieved using a Chrisofix splint for 2 weeks.

Twelve months postoperatively, a significant improvement in palmar tilt by 10° was observed in the patients under 12 years old who underwent surgery. In the 12-year-old patient, however, no radiological axis improvement was seen after 12 months.

## Results

In the analysis of all available English or German publications (*n* = 26) on Kirner deformity since 1985, a total of 121 cases were identified (Table 1). The average age of affected children was 9.36 years (± 2.6). Girls were significantly more affected than boys (F vs. M; 64% vs. 36%). In 56% of published cases, both hands were involved. In cases with unilateral involvement, 30% were affected on the right side and 13% on the left side. In all cases, the affected finger was the little finger. Only one publication by Sugiura reported involvement of the ring and middle fingers in addition to the little finger [6].

Where available, nearly all cases showed palmar curvature of the finger on X-ray as our presented case. Only in the publication by Lau was there a dorsal angulation, named as an inverse Kirner deformity [11].

**Fig. 5:**
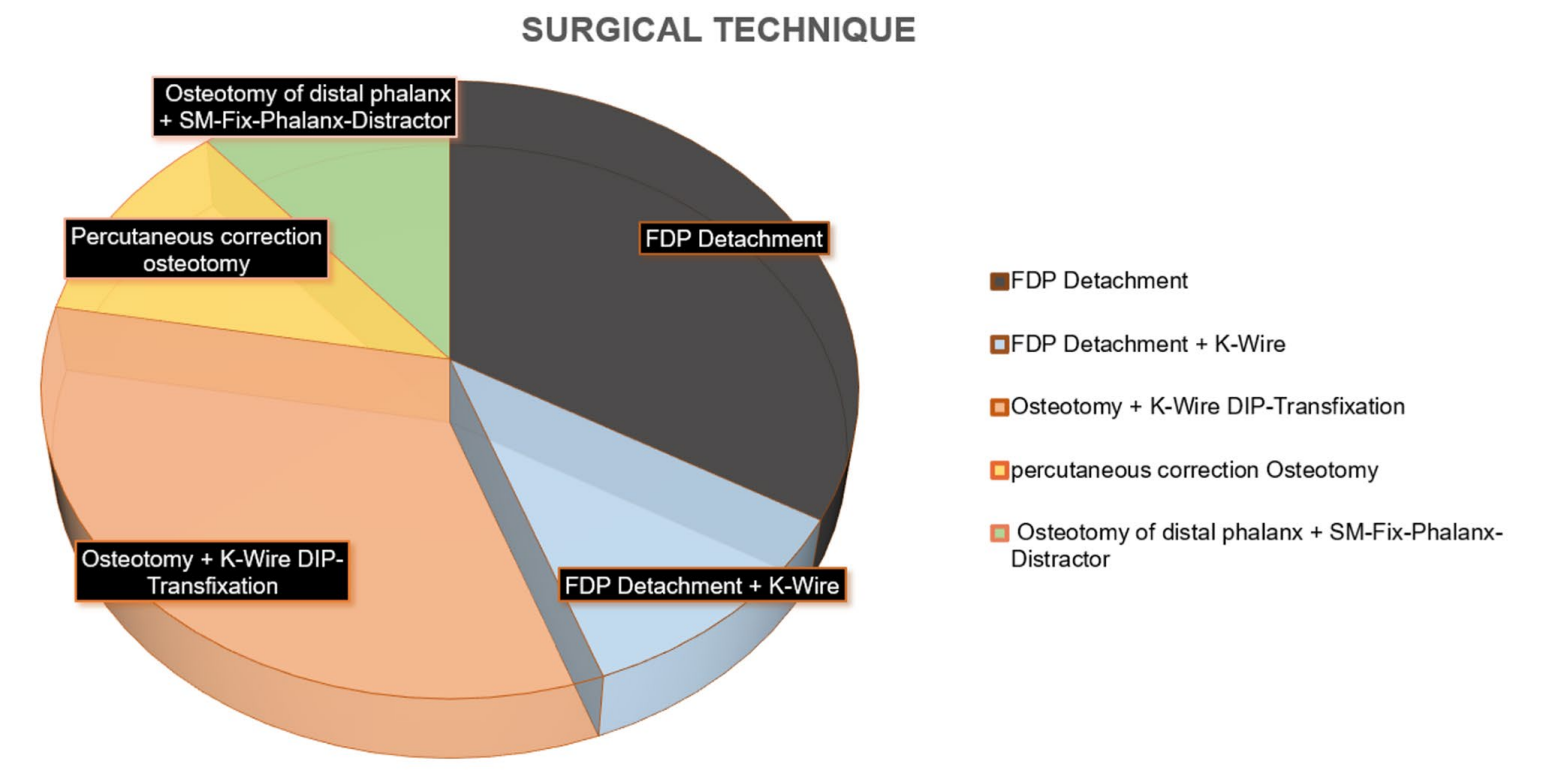
Surgical techniques

As mentioned earlier, various surgical treatment options are available. Across the included publications, a total of 9 surgeries were performed, accounting for 7.4% of all cases. One-third of the patients underwent a flexor digitorum profundus (FDP) detachment procedure. In one case, a K-wire transfixation of the distal interphalangeal joint was additionally performed to FDP detachment. Fairbank and colleagues performed three surgeries, which involved osteotomy and K-wire distal interphalangeal joint (DIP) transfixation. In another case, an osteotomy of the distal phalanx and the use of an SM-Fix phalangeal distractor were performed (Fig. 5).

In contrast, 92.5% of the patients did not undergo surgical treatment and were managed conservatively. Indications for surgical treatments were not mentioned clearly.

## Discussion

Primarily, it can be observed that the diagnosis of Kirner’s deformity is made through clinical examination and radiological imaging. Before planned surgical treatment, we recommend performing an MRI to determine the distal insertion of the flexor tendon for FDP detachment.

The prognosis of Kirner’s deformity is generally favorable. Since the deformity is typically asymptomatic, the quality of life of affected individuals is usually not impaired. This is reflected by over 90% of conservative cases found in our literature review. In cases where surgical correction was performed, the results are generally satisfactory. From our experience, surgical intervention before the age of 12 should be pursued for better functional and aesthetic outcomes. Long-term follow-up studies on patients have not yet been published.

Compared to the surgical method used by Carstam, we do not perform an osteotomy of the distal phalanx. Since, in our view, the change in the flexor tendon attachment is the cause, this eliminates a suspected cause of the palmar tilting due to the pull of the flexor tendon [12]. We also do not prefer temporary arthrodesis of the distal interphalangeal joint, as it leads to longer postoperative follow-up with intensive occupational therapy. This reduces costs and, on the other hand, allows for the earliest possible weight-bearing after complete wound healing.

Surgical procedures, such as partial tenotomy of the distal insertion of the flexor digitorum profundus tendon of the little finger, have been described to prevent abnormal growth stimuli. In the five cases we present, this surgical technique led to satisfactory outcomes, both functionally and aesthetically. Since the growth plate gradually closes with age, we recommend surgery before the age of 12 when indicated. Further studies are needed to determine the optimal age for surgery and its correlation with outcomes, as our case numbers are too small for statistically significant conclusions.

Kirner’s deformity is a rare, mostly asymptomatic anomaly of the distal phalanx of the little finger. Although the etiology is not fully understood, genetic predisposition likely plays a significant role. Genetic causes were not identified in the papers listed in Box 1. Further research is needed to better understand the genetic and developmental biology underlying Kirner’s deformity.

As previous case reports have indicated, female patients are more frequently affected than males, and a genetic link cannot be ruled out. The typical age range of 9 to 12 years aligns with the results of our literature review.

## Data Availability

No datasets were generated or analysed during the current study.
